# Proteomics for precision nutrition: current evidence and future directions

**DOI:** 10.1097/MCO.0000000000001226

**Published:** 2026-04-17

**Authors:** Mirko Marino, Cristian Del Bo', Patrizia Riso

**Affiliations:** Division of Human Nutrition, Department of Food, Environmental and Nutritional Sciences (DeFENS), Università degli Studi di Milano, Milan, Italy

**Keywords:** biomarkers, clinical translation, metabolic health, precision nutrition, proteomics

## Abstract

**Purpose of review:**

This review critically evaluates proteomics research applied to clinical nutrition and metabolism published between mid-2024 and early-2026, examining whether recent advances have moved the field closer to clinically actionable precision nutrition applications.

**Recent findings:**

Large prospective cohort studies show that circulating proteomic signatures reflect dietary patterns and are associated with incident cardiometabolic, hepatic, and neurodegenerative outcomes. In most analyses, these signatures capture plausible biological pathways, but their incremental predictive value beyond established risk models appears modest. Interventional studies confirm that circulating proteins respond to dietary modification, but these trials are considerably smaller than epidemiological cohorts and proteomic-guided randomized allocation has rarely been implemented to date. Although multiomics integration and machine-learning approaches have expanded discovery and improved pathway modeling, independent validation, cross-platform consistency, and clinically meaningful risk reclassification remain inconsistently demonstrated across studies.

**Summary:**

Diet–proteome associations are biologically coherent and reproducible at the population level. Nevertheless, translation into individualized dietary prescription remains to be demonstrated at scale. Robust evidence of cross-platform consistency, formal clinical utility, and outcome-driven trials incorporating proteomic-guided interventions will be key to enabling circulating proteomics to support routine precision nutrition practice.

## INTRODUCTION

Precision nutrition aims to refine dietary recommendations by accounting for measurable biological heterogeneity rather than relying exclusively on population-level averages (one-size-fits-all recommendations). Among the molecular approaches proposed to operationalize this paradigm shift, circulating proteomics has attracted particular interest. Plasma proteins integrate coordinated signals from metabolic regulation, inflammatory pathways, endocrine activity, vascular function, and tissue-specific stress responses, thereby providing a systemic readout within a single analytical layer. Recent reviews have documented the rapid expansion of high-throughput plasma proteomic technologies, while also underscoring their methodological constraints and translational challenges [1^▪▪^]. In particular, scalable affinity platforms, including aptamer-based assays (e.g., SomaScan) and antibody-based panels (e.g., Olink), have enabled cohort-scale quantification of thousands of plasma proteins, while making cross-platform calibration and shared reference standards increasingly important for translation. Against this backdrop, the present review examines human studies published between mid-2024 and early-2026 that explicitly link dietary exposures to circulating proteomic signatures and clinically relevant metabolic or disease outcomes. Rather than cataloguing associations, this review critically evaluates whether recent advances in diet–proteome research have generated evidence sufficient to support clinically actionable precision nutrition, or whether substantial methodological and translational gaps persist. 

**Box 1:**
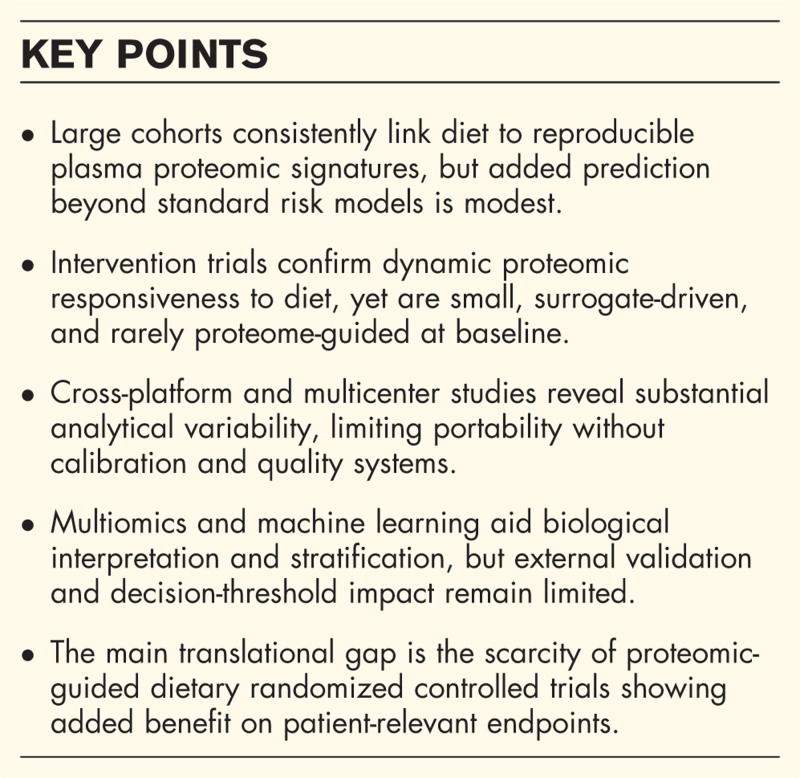
no caption available

## EPIDEMIOLOGICAL EVIDENCE

Over the past 18 months, large prospective cohorts have substantially expanded the evidence linking diet, circulating proteins, and disease risk. Nonetheless, most findings remain largely associative. The most compelling data derive from large-scale prospective studies. In an analysis including >50 000 participants, Zhu *et al.* [2^▪▪^] showed that plasma proteomic signatures aligned with healthy dietary patterns were associated with a lower risk of major chronic diseases and all-cause mortality within an event-driven framework. The scale, prospective design, and rigorous adjustment strategy position this study as a methodological reference point. Nevertheless, mediation analyses indicated that proteomic signatures explained only part of the observed diet–outcome relationship, suggesting biological relevance without full explanatory sufficiency. A comparable pattern was reported by Zhang *et al.* [3]. Using the EAT–Lancet dietary index, they found that circulating protein profiles were associated with incident heart failure. However, the incremental gains in discrimination beyond established risk models were generally modest. In both contexts, the proteome functioned as a statistically robust molecular intermediate readout, but its clinical utility beyond standard models was generally limited. Reproducible molecular correlates, therefore, do not necessarily translate into meaningful gains in risk stratification.

Parallel lines of investigation have examined diet–proteome relationship from an exposure-centered perspective. Du *et al.* [4^▪▪^] identified circulating protein signatures reflective of ultra-processed food intake and linked them to coronary heart disease, chronic kidney disease, and mortality, advancing the concept of molecular exposure metrics that complement self-reported intake and may mitigate measurement error. Zhao *et al.* [5^▪^] strengthened this approach by integrating metabolomic and proteomic signatures associated with ultra-processed food consumption and adverse liver outcomes, illustrating cross-omic convergence while retaining the inherent constraints of observational design. At greater dietary granularity, Du *et al.* [6^▪^] reported product- and fat-specific patterns within fermented dairy: cheese and low-fat fermented milk showed distinct protein signatures and inverse associations with all-cause mortality, whereas high-fat fermented milk was largely null. Similarly, Michaëlsson *et al.* [7^▪^] found that nonfermented versus fermented milk intake mapped to different cardiometabolic protein patterns (including ACE2/FGF21) and divergent signals for ischemic heart disease risk in women. These findings suggest that food processing and matrix characteristics may generate subtype-specific molecular fingerprints not fully captured by global dietary indices. Nutrient-level analyses have also been pursued. Solomon *et al.* [8^▪^] observed that higher intake of sodium was associated with measurable shifts in proteins related to inflammatory and vascular regulation, underscoring the feasibility for targeted nutrient–proteome investigations. In a large cohort setting, Sullivan *et al.* [9^▪^] reported cross-sectional proteomic correlates of vitamin D supplement use in ARIC, providing micronutrient-linked molecular associations rather than causal supplementation effects. Plant-based dietary patterns, operationalized either as diet groups defined by animal-food consumption or as plant-based diet indices, provide one of the most consistent examples of systemic proteomic remodeling. In this context, Tong *et al.* [10^▪▪^] documented coordinated shifts across nearly 3000 proteins in a cohort approaching 50 000 individuals. Complementary work using index-derived plant-based signatures linked plant-based adherence to cardiovascular events via proteomic mediators [11^▪^], while large prospective analyses extended index-based associations to lung cancer risk [12^▪^]. In addition, Lim *et al.* [13^▪^] identified pathway-level circulating proteins linking diet-quality indices to incident type 2 diabetes, implicating inflammatory and metabolic intermediates. Yang *et al.* [14^▪^] further extended diet–proteome associations to incident dementia through proteins related to vascular and synaptic biology. Across these independent cohorts and outcome domains, the molecular response appears internally coherent and reproducible. Nonetheless, effect sizes are generally moderate, mediation remains partial, and formal reclassification metrics rarely reach thresholds likely to change clinical management, preventive counseling, or the targeting of dietary interventions. The observational evidence is therefore biologically consistent and increasingly broad in scope, but its translation into measurable clinical impact remains underdeveloped.

## INTERVENTIONAL EVIDENCE

Evidence from structured dietary interventions further supports the biological responsiveness of the circulating proteome. Within the DIRECT-PLUS framework, Zelicha *et al.* [15] reported that greater adherence to a green Mediterranean diet was associated with adiposity-related proteomic shifts, while Goldberg *et al.* [16^▪^] linked reductions in MRI-assessed intrahepatic fat to coordinated changes in circulating protein patterns. Complementing these findings, El-Sabawi *et al.* [17^▪^] described temporal proteomic remodeling paralleling improvements in hepatic steatosis during metabolic intervention. Collectively, these studies indicate that circulating proteins track organ-level metabolic change under controlled dietary modification. However, their design contrasts with that of large epidemiological cohorts. Sample sizes are smaller, follow-up is shorter, and event counts are insufficient to evaluate hard clinical endpoints. Importantly, these trials employed proteomics primarily as a mechanistic readout, an essential prerequisite for panel selection and biological validation, but not yet as a stratification tool to guide allocation. Energy restriction and aging-related interventions similarly demonstrate molecular plasticity without establishing clinical utility. Cagigas *et al.* [18^▪^] reported reversal of aging-associated proteomic signatures following short-term severe energy restriction in adults with prediabetes, suggesting reversibility of systemic molecular aging signals. Despite this biological coherence, these studies remain limited in scope. Most include fewer than 300 participants, rely on surrogate or imaging-based endpoints, and lack long-term outcome validation. To date, baseline proteomic profiling has not been tested as an allocation tool in trials. Thus, temporal responsiveness is robust, but clinical translation now hinges on trials that use baseline proteomic profiles to assign interventions, with preregistered thresholds and patient-relevant endpoints to quantify incremental benefit.

## MECHANISTIC REFINEMENT AND PHENOTYPIC SUBTYPING

Beyond population-level associations, several studies have moved toward tissue-level interpretation by examining organ-specific phenotypes and extracellular vesicle (EV) proteomics. Pirrotte *et al.* [19^▪^] identified distinct EV-derived proteomic signatures associated with hepatic steatosis in postmenopausal women, proposing that EV cargo may capture more tissue-proximal information than bulk plasma proteomics. This work reflects a broader conceptual shift. Rather than treating circulating proteins solely as diffuse systemic markers, EV-based analyses aim to isolate signals that more directly reflect organ-specific biology. However, this increased granularity entails methodological trade-offs. EV isolation strategies, centrifugation workflows, and purity assessments vary considerably across laboratories, and standardized protocols remain limited. As a result, cross-study comparability is constrained, and independent external validation remains scarce. Extensions of diet–proteome associations into organ-specific settings have followed a similar trajectory. Rovira *et al.* [20^▪^] explored pathway-level proteomic alterations in advanced chronic kidney disease (CKD) in the context of Mediterranean diet patterns, while Yang *et al.* [21^▪^] and Lu *et al.* [22^▪^] linked adherence to EAT-Lancet diet with incident CKD and chronic obstructive pulmonary disease (COPD) through circulating protein signatures. These studies broaden the clinical scope of proteomic research into renal and pulmonary domains, reinforcing the notion that circulating proteins capture multiorgan biological signals. Nevertheless, the findings remain associative and do not establish whether baseline proteomic phenotypes can identify patients more likely to benefit from specific dietary interventions within these disease settings.

Mechanistic resolution has also progressed through higher-dimensional metabolic profiling. Kolic *et al.* [23] identified proteomic predictors of nutrient-specific insulin secretion, suggesting that circulating proteins may reflect heterogeneity in postprandial metabolic responses. Kjærgaard *et al.* [24^▪▪^] further delineated personalized molecular signatures of insulin resistance and type 2 diabetes using integrated proteomic and phosphoproteomic approaches, pointing toward molecular subtypes within broadly defined metabolic phenotypes. Such analyses enhance pathway-level interpretation and strengthen biological plausibility. Yet, the transition from molecular subtyping to therapeutic stratification has not been realized. No study has prospectively allocated dietary interventions based on these molecular classifications to determine whether subtype-informed strategies improve clinical outcomes. Thus, although mechanistic depth and phenotypic refinement have increased, evidence supporting intervention-level stratification remains limited.

## MULTI-OMICS AND COMPUTATIONAL INTEGRATION

The integration of proteomics with additional molecular layers has become a defining feature of recent diet–proteome research. Rather than treating circulating proteins as isolated biomarkers, several investigations have embedded them within broader systems architectures. Deng *et al.* [25^▪^] combined serial serum proteomics with gut microbiome profiling to model cardiometabolic trajectories associated with EAT-Lancet adherence, demonstrating that coordinated shifts in microbial composition and protein abundance track dietary behavior over time. Within the Framingham Heart Study, Lee *et al.* [26^▪^] moved beyond single-protein analyses by applying network-based modeling to delineate interconnected clusters linking dietary patterns to intermediate cardiometabolic traits, thereby shifting the focus toward pathway-level organization rather than isolated biomarkers. Fu *et al.* [27^▪^] advanced this systems perspective by embedding proteomic signatures within broader multiomics lifestyle clusters, illustrating how healthy and unhealthy behavioral phenotypes are mirrored by coordinated molecular architectures spanning both proteomic and metabolomic layers. In parallel, Liang *et al.* [28^▪^] and Kim *et al.* [11^▪^] connected circulating protein patterns to incident type 2 diabetes and cardiovascular events across diverse populations, lending additional plausibility to multilayer mediation frameworks.

The expanding application of machine learning has further increased analytical flexibility, but not without trade-offs. Chen *et al.* [29^▪^] used computational optimization to explore diet-based strategies for dementia risk reduction, integrating proteomic analyses as a downstream mechanistic readout, while Navratilova *et al.* [30^▪^] identified AI-derived food preference clusters associated with distinct metabolomic and proteomic profiles. Khawaja *et al.* [31^▪^] highlighted the utility of machine-learning pipelines for feature refinement in high-dimensional proteomic data. Collectively, these studies signal a shift from conventional biomarker discovery toward integrative modeling approaches. However, greater algorithmic sophistication does not by itself guarantee stronger clinical reliability. In several analyses, the ratio of clinical events to modeled variables remains relatively low, increasing susceptibility to overfitting. Validation restricted to a single biobank does not ensure transportability across populations or analytical platforms. Reported improvements in discrimination metrics, including incremental gains in area under the curve (AUC), are often modest and unlikely to alter clinical decision thresholds. Furthermore, signatures derived from affinity-based platforms may not be reproducible when quantified using mass spectrometry, thereby limiting cross-platform generalizability. Without disciplined feature reduction, calibration across technologies, and replication in independent cohorts, proteomic models may remain optimized for the development dataset, limiting clinical transferability. In this context, integrative modeling enriches biological interpretation while simultaneously exposing methodological limitations that must be resolved before meaningful translation can occur.

## METHODOLOGICAL CHALLENGES

Technical variability remains a major translational barrier in the application of circulating proteomics to nutrition research. A large cross-platform evaluation illustrates how analytical concordance can be fragile. In a comparison of >2000 plasma proteins measured using two high-throughput affinity platforms in 4000 individuals, Wang *et al.* [32^▪▪^] reported that only a subset of proteins demonstrated strong agreement across assays. Discrepancies were observed not only in absolute concentrations but also in rank ordering, directly challenging the portability of proteomic signatures discovered within a single technological ecosystem. A related concern emerges from the multicenter longitudinal quality assessment conducted by Kardell *et al.* [33^▪▪^], who documented measurable inter-laboratory variability in mass spectrometry–based plasma proteomics despite harmonized procedures. Differences in instrument calibration, sample preparation workflows, and batch structure were sufficient to introduce variability that could meaningfully affect downstream modeling. These practical sources of variation can materially affect downstream modeling. Most diet–proteome investigations rely on one analytical platform and pipeline, with limited replication across technologies. Preanalytical variability further complicates interpretation. Fasting status, timing of blood collection, anticoagulant choice, storage conditions, freeze–thaw cycles, and long-term biobanking practices all influence measured protein abundance, yet reporting of these parameters is inconsistent across dietary studies. In interventional settings, promoting improved adherence to the Mediterranean diet [15,16^▪^] or caloric restriction [18^▪^], proteomic analyses are often performed in subsets of participants and may not uniformly document sample handling across centers. Extracellular vesicle–based approaches [19^▪^] introduce additional layers of variability, including vesicle isolation protocols, centrifugation speeds, and purity assessment strategies that are rarely standardized. Shared reference materials, inter-laboratory proficiency testing, and cross-platform calibration are still lacking. As a result, proteomic scores may remain site- and platform-dependent, limiting transferability, rather than stable biomarkers transferable across studies.

Beyond technical instability lies an equally important conceptual issue, association, mediation, and causality are sometimes treated interchangeably. Large cohort analyses [2^▪▪^,^4^^▪▪^,^10^^▪▪^,11^▪^,28^▪^] demonstrate independent associations between dietary patterns, circulating protein signatures, and incident outcomes. Some incorporate statistical mediation models to estimate the proportion of the diet–disease relationship explained by selected proteins. Even pathway-focused analyses, such as those linking diet-quality indices to incident type 2 diabetes through specific inflammatory and metabolic proteins [13^▪^], remain dependent on mediation assumptions that are difficult to fully verify in observational settings. Mediation analysis in observational settings rests on strong assumptions, notably the absence of unmeasured confounding between mediator and outcome and correct specification of model structure. These assumptions are often difficult to verify in nutritional epidemiology. Consequently, proteomic signatures may function as refined correlates or composite risk indicators rather than as confirmed biological intermediates. Formal causal inference approaches, such as Mendelian randomization using valid genetic instruments for specific proteins, have not yet been systematically embedded within diet–proteome–outcome frameworks during the reviewed period. Intervention studies further highlight this gap. Although the circulating proteome clearly responds to dietary change [15,16^▪^,18^▪^], proteomics has not yet been used to assign dietary strategies at baseline, consistent with a field still moving from phenotype discovery and replication toward stratified intervention testing. Overall, the evidence is strong for association and biological plausibility, and intervention studies confirm proteomic responsiveness to diet, but causal interpretation and decision utility remain limited. Preserving clear distinction among association, mediation, and causation, and transparently reporting assumptions, validation strategies, and analytical limitations, will be essential if diet–proteome research is to advance beyond sophisticated molecular description toward actionable clinical implementation.

## TRANSLATIONAL THRESHOLD

**FIGURE 1:**
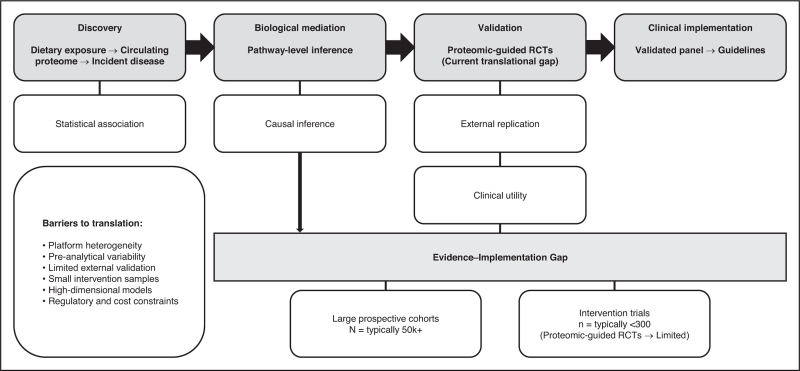
Translational architecture of circulating proteomics in precision nutrition. Conceptual framework outlining the sequential stages from proteomic discovery to clinical implementation in diet–proteome research. The upper tier depicts the progression from identification of circulating proteomic signatures (discovery) through biological mediation and causal inference, to external validation and eventual implementation. The central band represents the evidence–implementation gap separating statistically robust associations from demonstrated clinical utility. The left panel summarizes the principal methodological constraints identified in recent human studies, including cross-platform discordance, preanalytical variability and batch effects, limited external replication, small sample sizes in intervention trials, and vulnerability to machine-learning overfitting. The lower tier illustrates the asymmetry of the evidence base, contrasting large prospective cohorts with substantially smaller dietary intervention trials, and the absence of proteomic-guided randomized controlled trials demonstrating hard outcome benefit. The framework delineates criteria required for translational validity and implementation in targeted nutrition approaches. EV, extracellular vesicles; LC–MS, liquid chromatography–mass spectrometry; ML, machine learning; RCT, randomized controlled trial.

For circulating proteomics to be credibly integrated into precision nutrition, the evidentiary standard likely needs to extend beyond statistical association (Fig. 1). Prospective associations with hard endpoints are increasingly supported in large cohorts. The key question is whether proteomic information adds decision-relevant utility beyond standard predictors. In most studies, gains in discrimination and reclassification are modest, and statistical significance is sometimes interpreted as clinical usefulness. Translation also requires that signatures behave as portable measurements, not study-specific artifacts. This implies locked panels, transparent calibration procedures, and performance that remains stable across laboratories, platforms, and processing workflows, conditions that are prerequisites for any clinically interpretable risk tool. The decisive translational test, however, remains prospective stratification. The use of baseline proteomic profiles to guide the allocation of dietary strategies and then quantifying incremental benefit on patient-relevant endpoints is a crucial target. To date, dietary intervention studies have convincingly demonstrated proteomic responsiveness and pathway engagement, but they have not yet evaluated proteomics as an allocation variable with prespecified decision rules. Even emerging personalization frameworks incorporating multiomics data generally use proteomics for characterization rather than as the engine of stratified randomization. Progress beyond the current stage will require disciplined panel reduction to signatures demonstrably stable across technologies, replication in diverse populations with transparent reporting of calibration and performance metrics, explicit evaluation of decision utility (not association alone), and rigorously designed stratified randomized trials directly comparing proteomic-guided nutrition with guideline-based dietary care.

## CONCLUSION

Large human studies now demonstrate that dietary patterns leave reproducible imprints on the circulating proteome and that these signatures are associated with incident cardiometabolic and other clinical outcomes. Interventional trials confirm proteomic responsiveness to dietary modification, but it has so far served mainly as a mechanistic or response readout rather than a tool for baseline allocation. At present, proteomics mainly enhances risk characterization, with limited evidence of added decision utility beyond standard predictors. Crossing the translational threshold will require cross-platform harmonization, independent replication in diverse populations, and adequately powered randomized trials designed to test whether proteomic-guided dietary strategies improve hard clinical endpoints compared with guideline-based recommendations.

## Acknowledgements

*Authors acknowledge the OnFoods project funded under the National Recovery and Resilience Plan (NRRP), Mission 4 Component 2 Investment 1.3 – Call for proposals No. 341 of 15 March 2022 of Italian Ministry of University and Research funded by the European Union – NextGenerationEU; Award Number: Project code PE00000003, Concession Decree No. 1550 of 11 October 2022 adopted by the Italian Ministry of University and Research, CUP D93C22000890001, Project title “ON Foods – Research and innovation network on food and nutrition Sustainability, Safety and Security – Working ON Foods”.*

### Financial support and sponsorship

*None.*

### Conflicts of interest

*There are no conflicts of interest.*

## REFERENCES AND RECOMMENDED READING

Papers of particular interest, published within the annual period of review, have been highlighted as:

▪ of special interest
▪▪ of outstanding interest
